# Is Beta Ketothiolase Deficiency an Uncommon Disease or an Unsuspected Diagnosis? The Role of Genetic Biochemistry Approaches in Metabolic Acidosis

**DOI:** 10.3390/pediatric17060113

**Published:** 2025-11-03

**Authors:** Luis D. Campos-Acevedo, Joel Arenas-Estala, Marisol Ibarra-Ramírez, Graciela A. López-Uriarte, María C. Ruíz-Herrera, Marcelo Rodríguez-Rivera, Laura E. Martínez-de-Villarreal

**Affiliations:** Genetics Department, Hospital Universitario “Dr. José Eleuterio González” and Medicine Faculty, Autonomous University of Nuevo León, Monterrey 64460, Mexico; lcamposa@uanl.edu.mx (L.D.C.-A.); joel.arenase@uanl.edu.mx (J.A.-E.); mibarrar@uanl.edu.mx (M.I.-R.); glopezu@uanl.edu.mx (G.A.L.-U.); maria.ruiz@genetica-uanl.mx (M.C.R.-H.); marcelo.rodriguez@genetica-uanl.mx (M.R.-R.)

**Keywords:** metabolic acidemia, ketolysis defects, isoleucine metabolism

## Abstract

Beta ketothiolase deficiency is a hereditary metabolic disorder caused by the pathogenic variants of the *ACAT* gene, which encodes for the mitochondrial enzyme acetoacetyl-CoA thiolase. Patients with a deficiency of the enzyme experience recurrent episodes of metabolic ketoacidosis. Knowledge of the clinical course of this entity, together with the available diagnostic tests, allows for its early diagnosis and prompt intervention to avoid complications or death of the infant. In this study, we present a case of a 9-month-old girl that attended the emergency room and diagnosis was made at the first episode of metabolic ketoacidosis.

## 1. Introduction

**Figure 1:**
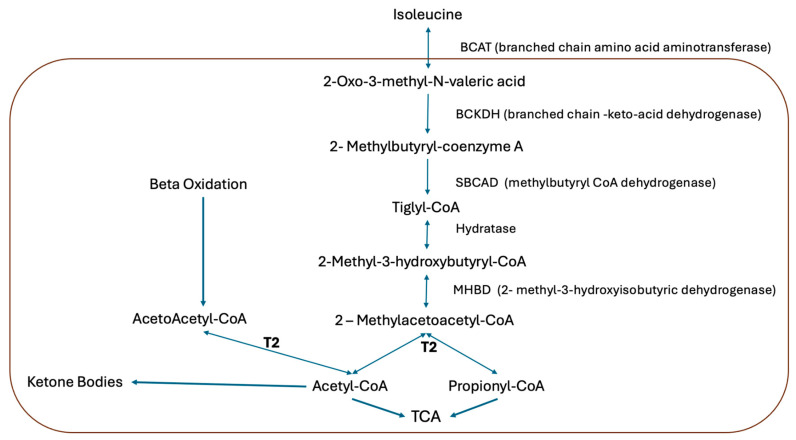
Isoleucine catabolic pathway. The acetoacetyl-CoA thiolase (T2) is involved in isoleucine catabolism, hepatic ketogenesis, and ketolysis in extrahepatic tissues [4].

Beta ketothiolase deficiency is a hereditary metabolic disorder with an autosomal recessive inheritance trait. It is caused by the pathogenic variants of the *ACAT* gene, which encodes for the mitochondrial enzyme acetoacetyl-CoA thiolase (T2) [1,2]. This enzyme plays an important role in the metabolism of the amino acid isoleucine and metabolism of the ketone bodies, catalyzing the conversion of acetoacetyl-CoA into two acetyl-CoA molecules [2,3]. It is a disorder of ketolysis characterized by the accumulation of organic acids. Along with 3-hydroxy-3-methylglutaryl-CoA dehydrogenase deficiency, T2 deficiency is one of the most common causes of ketolysis disorders (Figure 1).

Diagnosis in these patients is challenging, as their clinical presentation can be confused with other, more common conditions such as sepsis, prolonged fasting and diabetic ketoacidosis. Patients with acetoacetyl-CoA thiolase deficiency experience recurrent episodes of metabolic ketoacidosis that usually begin between 6 and 18 months of age. They may be precipitated by fasting, infections, or metabolic stress states, and manifest with vomiting, lethargy, hypotony and, in severe cases, coma [5,6]. During attacks, high anion gap metabolic acidosis (>20), occasionally hypoglycemia or normoglycemia, and severe ketonuria may occur.

To date, very few cases of this disorder have been reported, with its estimated incidence being less than 1 per million newborns [7]. Neonatal screening programs using tandem mass spectrometry (MS/MS) include early detection of this disease and 2-methyl-3-hydroxybutyryl-CoA dehydrogenase deficiency through the determination of tiglyl-carnitine (C5:1), which has led to the identification to a greater number of patients [8,9,10,11]. However, not all newborns are screened and false negative results can also occur. Therefore, it is necessary to understand the clinical picture of this condition, in order to recognize it, request the appropriate test to make an early diagnosis, and provide timely intervention to reduce the comorbidities that may occur.

The determination of urinary organic acids by gas chromatography/mass spectrometry (GC/MS) in addition to tandem spectrometry (MS/MS) comprises a workflow that allows for the diagnosis of organic acidemias.

In this study, a case of T2 deficiency is presented in which the suspicion of the condition and the availability of the necessary laboratory tests allowed for the diagnosis during the first crisis, with the aim of alerting emergency unit physicians to this condition.

## 2. Case Presentation and Methods

A 9-month-old female was admitted to the emergency department with hyaline rhinorrhea and cough, followed by multiple episodes of vomiting, irritability, food refusal, weakness, flaccidity, and signs of respiratory distress. She was the first child of non-consanguineous parents with no relevant family history and had been born at term by vaginal delivery, with weight, length, and head circumference within normal parameters for gestational age. Growth and development had been appropriate until presentation.

During evaluation in the emergency department, she developed Kussmaul respiration and altered consciousness. Chest X-ray revealed a perihilar reticular interstitial pattern compatible with viral pneumonia. At admission, blood gas analysis showed severe metabolic acidosis with pH 6.81 (reference 7.35–7.45), bicarbonate 3.2 mmol/L (22–26 mmol/L), sodium 137.1 mmol/L, potassium 4.6 mmol/L, chloride 108.4 mmol/L, PCO_2_ 20 mmHg, and PO_2_ 76 mmHg, with an elevated anion gap of 30 mmol/L (8–16 mmol/L). Lactate was 1.3 mmol/L (0.5–2.2 mmol/L) and ammonia 55 µmol/L (10–50 µmol/L). Blood glucose was 90 mg/dL, which allowed diabetic ketoacidosis to be ruled out. Renal function parameters (creatinine 0.7 mg/dL, urea 6 mg/dL) were within normal limits, with no evidence of acute kidney injury. Bacterial cultures (blood, urine, tracheal aspirate) were negative, and inflammatory markers did not support systemic infection. Urinalysis revealed ketones >160 mg/dL (+++). Accordingly, a diagnosis of high anion gap metabolic acidosis with marked ketonuria was established.

Initial management by the pediatric team focused on general supportive care, including intubation, fluid resuscitation, bicarbonate infusion, and intravenous glucose to reverse catabolism, but no improvement was observed. The pediatric evaluation had already ruled out the most common acquired causes due to the absence of hyperglycemia, lack of evidence of systemic infection, and absence of a history of prolonged fasting or drug exposure. The severity of the metabolic acidosis with ketonuria in a young child, together with the clinical response to anti-catabolic measures, raised the suspicion of an inborn error of metabolism. For this reason, an early consultation with the Genetics Department was requested. Physical examination revealed no craniofacial dysmorphisms or characteristic odor.

## 3. Results

Determination of urinary organic acids via GC/MS and acylcarnitine profile via MS/MS were requested. The results of the metabolic studies showed elevated urinary levels of 3-hydroxybutyric acid, 2-methyl-3-hydroxybutyric acid, 3-hydroxi isovaleric acid, tiglilglycine, and lactic acid, and elevated ratios of C3DC + C4OH = 0.47 mmol/L (<0.27 mmol/L) and C3DC + C4OH/C5DC + C6OH = 3.22 mmol/L (<1.8 mmol/L). A diagnosis of beta-ketothiolase deficiency was established and confirmed via next-generation sequencing (NGS), identifying two variants of the *ACAT1* gene: one pathogenic c.473A>G (p.Asn158Ser) and another not previously reported, c.826 + 3_826 + del (intron affecting the splicing site. In silico predictors classified it as deleterious).

A pediatric nutritionist indicated dietary isoleucine restriction, and management with levocarnitine was initiated.

## 4. Discussion

Beta-ketothiolase deficiency should be considered when pediatric patients present with an acute attack of metabolic acidosis after prolonged fasting, infections of the digestive or respiratory tract, or any febrile condition. Patients with severe ketosis or metabolic acidosis, even in the postprandial state, tend to lack a characteristic urinary organic acid profile, which can hamper their identification via routine chromatography. Both disorders can manifest from the neonatal period and require enzymatic and/or molecular studies for definitive differentiation [2].

Another relevant condition is 2-methyl-3-hydroxybutyryl-CoA dehydrogenase deficiency (MHBD)—a congenital disorder of isoleucine catabolism that, in isolated cases, simulates both the clinical picture and the urinary organic acid profile characteristic of BKT deficiency; particularly with elevated 2-methyl-3-hydroxybutyrate (2M3HB) and tiglilglycine (TIG) levels.

However, MHBD is an X-linked disorder with a progressive neurodegenerative course and, unlike BKTD, it does not show elevated 2-methylacetoacetate (2MAA), even after an oral isoleucine overload. T2 enzyme activity is normal in these cases, allowing for differential diagnosis using specific tests [2].

Monocarboxylate transporter type 1 (MCT1) deficiency—a defect in ketone utilization caused by mutations in the *SLC16 A* gene—has recently been described as a differential diagnosis. This entity may present with recurrent episodes of ketoacidosis of apparently unexplained cause, with variable levels of ketonuria and generally normal blood pH between attacks. Its identification requires targeted genetic analysis, especially in patients without a clear diagnosis [2].

In the clinical context of ketoacidosis with hyperglycemia, diabetic ketoacidosis should also be considered, although blood glucose levels in this disorder are typically much higher than those seen in BKTD. In such cases, determination of glycated hemoglobin may be useful to distinguish between these two conditions. Similarly, in children with moderate hypoglycemia and hyperketonemia, the condition could correspond to functional ketotic hypoglycemia—a benign and self-limited condition. Meanwhile, in BKTD, metabolic acidosis is usually more severe. Measuring the ratio of free fatty acids to total ketone bodies may aid in differentiation as, in ketone utilization defects, this ratio is usually less than 0.3 mmol/L in the early stages of fasting [2].

Other disorders that should be considered include defects in glucose or glycogen metabolism, such as glycogen synthase deficiency, which can also present with hypoglycemia and ketosis. In these cases, carbohydrate metabolism studies and liver tests can provide relevant information. Finally, when ketoacidosis is accompanied by hyperammonemia, other organic acidemias should be ruled out; these can be differentiated through a detailed analysis of organic acids in urine. Furthermore, if the possibility of congenital lactic acidosis is suspected, measuring lactate and pyruvate levels during the first attack is recommended.

In our case, the diagnosis was made based on the suspicion of a metabolic disorder and as the determination of urine organic acids revealed elevated levels of 2-methyl-3-hydroxybutyrate, 2-methylacetoacetate, and tiglilglycine, while the blood acylcarnitine profile revealed elevated tiglylcarnitine [7,8].

The molecular study demonstrated a compound heterozygous mutation in the *ACAT1* gene. More than 70 pathogenic variants in the *ACAT1* gene resulting in beta-ketothiolase deficiency have been identified. There is no clear correlation between the genotype and the severity of the clinical manifestations. Described mutations include deletions, insertions, and point variants in various regions of the gene [1,2]. Recurrent pathogenic variants have been reported in specific populations, such as p.Arg208* in Vietnam, while others have been identified in isolated sporadic cases [7].

## 5. Conclusions

This is the first case reported in a Mexican patient. The diagnosis during the initial crisis was achieved through a combination of high clinical suspicion and the availability of advanced metabolic and genetic testing at our medical center. Developing an algorithm that includes metabolic and genetic testing of patients presenting with acidemias with no apparent cause is expected to allow for the identification of a greater number of cases.

## Data Availability

The original contributions presented in this study are included in the article. Further inquiries can be directed to the corresponding author(s).

## Abbreviations

The following abbreviations are used in this manuscript:

T2	Enzyme acetoacetyl-CoA thiolase
BKTD	Beta ketothiolase deficiency
MS/MS	Tandem mass spectrometry
GC/MS	Gas chromatography/mass spectrometry
NGS	Next-generation sequencing
MHBD	2-methyl-3-hydroxybutyryl-CoA dehydrogenase deficiency
TIG	Tiglilglycine
2MAA	2-methylacetoacetate
MCT1	Monocarboxylate transporter type 1
